# Physical activity may help mitigate sleep‐related cognitive deficits in older adults

**DOI:** 10.1002/alz70860_098137

**Published:** 2025-12-23

**Authors:** Kelsey R. Sewell, Audrey M. Collins, Jeremiah J Peiffer, Belinda M. Brown, Kirk I. Erickson, The IGNITE Consortium

**Affiliations:** ^1^ Advent Health, Orlando, FL, USA; ^2^ AdventHealth Research Institute, Orlando, FL, USA; ^3^ Murdoch University, Perth, Western Australia, Australia; ^4^ Edith Cowan University, Perth, Western Australia, Australia

## Abstract

**Background:**

Physical activity and sleep are both related to brain health, and these lifestyle factors also share a bi‐directional relationship. However, in the context of cognitive function, sleep and physical activity are seldom considered together in cross‐sectional studies, and the influence of each lifestyle factor in the context of interventions (e.g., exercise) remains unclear. The purpose of this research is to investigate whether sleep and physical activity may interact to influence brain health in older adults.

**Method:**

This abstract will present data from two studies. The first is a previously unpublished cross‐sectional study in healthy older adults (*n* = 589; 69.8 ±3.7 years). Sleep was measured via the Pittsburgh Sleep Quality Index (PSQI) and both sleep and physical activity were measured via 24‐hour actigraphy for 7 days. We investigated the moderating influence of physical activity on associations between sleep and cognitive function in five domains. Secondly, we will present published data from a randomized controlled trial which was a 6‐month, supervised exercise intervention in 89 cognitively unimpaired older adults (68.76±5.32 years). We investigated the influence of baseline sleep, measured by PSQI, on exercise‐induced cognitive improvement across the course of the intervention.

**Result:**

Cross‐sectionally, moderate‐to‐vigorous physical activity (MVPA) moderated the association between self‐reported sleep efficiency and episodic memory, processing speed, EF/attentional control, and working memory (*β*_[range]_ = ‐0.10 – ‐0.17, all *p* < .05). Light physical activity moderated the association of actigraphy‐measured WASO with EF/attentional control and processing speed (*β*_s_ = 0.10, all *p* < .05). The direction of these results was such that the association between lower sleep efficiency and greater WASO with poorer cognitive performance was stronger in those with low physical activity levels. From our exercise intervention study, we found that that those with poorer sleep efficiency at baseline showed the greatest exercise‐induced improvements in episodic memory from pre‐ to post‐intervention (β=−0.024, *p* = 0.004).

**Conclusion:**

Our results suggest that physical activity and sleep interact to influence cognitive function, and the efficacy of exercise interventions to improve cognition may be influenced by sleep. Taken together, our data suggest that physical activity may compensate for some negative influences of poor sleep on cognition.

